# “Shell Shock” Not a Medical Term

**Published:** 1918-09

**Authors:** 


### "SHELL SHOCK" NOT A MEDICAL TERM

Apropos of the editorial comment on "No More Shell Shock" in the August number of War Medicine is the announcement from the Division of Neurology and Psychology that "shell shock" has no place in the nomenclature of diseases among the soldiers of the American Expeditionary Forces. In a recent bulletin the Senior Consultant in Neuro-Psychiatry says that in spite of the fact that the circular regarding "Sick and Wounded" Reports states clearly that the term "shell shock" will not be accepted as a diagnosis of disability or death, it is still being used by medical officers in many tactical divisions and base hospitals.

The prevalence of the war neuroses in an army is in our own hands nearly as much as the control of infectious diseases. There are measures which tend to suggest functional nervous diseases and there are others which tend to prevent them. It is the duty of medical officers to try to prevent mental and nervous diseases as well as communicable ones, and there is no excuse for suggesting permanent or semi-permanent nervous invalidism to a man suffering from concussion or exhaustion by using the term "shell shock" on his diagnosis tag or field medical card.

If the medical officer thinks that a man has been concussed or is physically exhausted he should say so, and, if he thinks that the soldier is suffering more from nervousness than from concussion or exhaustion, he should indicate the fact by using the terms provided in the nomenclature of diseases, or the symbol "N. Y. D." followed by "nervous" in parentheses. Many a good soldier is being lost to the front lines through carelessness in this respect, for few men can withstand the suggestion of disease when that suggestion is made by those whom they rightly regard as authorities in medical matters.

The control of the war neuroses is one of the great new medical problems of this war. Those directly responsible for dealing with the problem are working hard to minimize these disorders in the A.E.F., but they need the help of every company and regimental medical officer and every man on the staff of a base hospital. No medical officer should use the term "shell shock" either in reports or in conversation, nor should nurses and hospital corps men be permitted to use it.

It is not a medical term but a piece of military slang which applies to a large group of conditions; the name is descriptive of only a small proportion of all cases. It is not permitted in the British or French armies or in the armies of our enemies.

To discontinue using the term "shell shock" shows a desire to preserve man-power, to be accurate and clear in medical nomenclature and to follow regulations framed after thoughtful consideration; to continue using it shows clinical slovenliness, disregard for regulations, and indifference to the preventable wastage of man-pover. This is a little thing but so was the nail, for the lack of which the horseshoe, the horse, the rider and a kingdom were lost.

